# Hypoxia-Inducible Factor (HIF) as a Target for Novel Therapies in Rheumatoid Arthritis

**DOI:** 10.3389/fphar.2016.00184

**Published:** 2016-06-27

**Authors:** Susan Hua, Thilani H. Dias

**Affiliations:** ^1^School of Biomedical Sciences and Pharmacy, University of NewcastleCallaghan, NSW, Australia; ^2^Hunter Medical Research InstituteNew Lambton Heights, NSW, Australia

**Keywords:** rheumatoid arthritis, hypoxia, hypoxia-inducible factor, inflammation, synovitis, angiogenesis, cartilage degradation, targeted therapy

## Abstract

Hypoxia is an important micro-environmental characteristic of rheumatoid arthritis (RA). Hypoxia-inducible factors (HIF) are key transcriptional factors that are highly expressed in RA synovium to regulate the adaptive responses to this hypoxic milieu. Accumulating evidence supports hypoxia and HIFs in regulating a number of important pathophysiological characteristics of RA, including synovial inflammation, angiogenesis, and cartilage destruction. Experimental and clinical data have confirmed the upregulation of both HIF-1α and HIF-2α in RA. This review will focus on the differential expression of HIFs within the synovial joint and its functional behavior in different cell types to regulate RA progression. Potential development of new therapeutic strategies targeting HIF-regulated pathways at sites of disease in RA will also be addressed.

## Introduction

**Figure 1:**
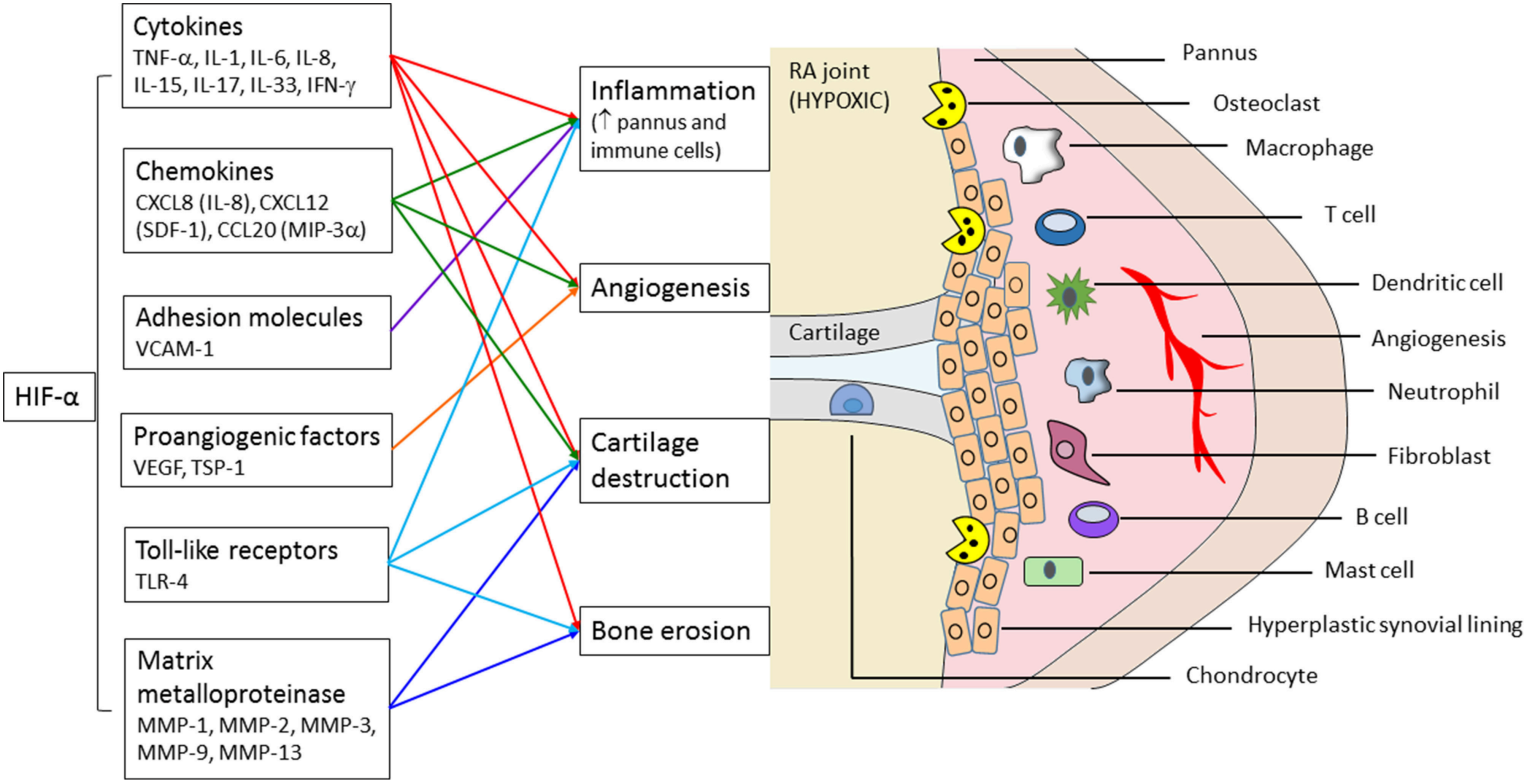
**Role of hypoxia-regulated HIF-α transcription factors in inflammation and destruction of the RA joint**. HIF-α can directly modulate the expression of mediators that are involved in synovitis, angiogenesis, cartilage destruction, and bone erosion. This can also influence the composition of the cellular infiltrate in the synovial tissue and perpetuate the progression of disease.

Rheumatoid arthritis (RA) is the most common form of inflammatory arthritis. It is an autoimmune and polyarthritic condition, characterized by inflammation of the synovium (synovitis), progressive cartilage destruction, and bone erosion, which ultimately results in loss of integrity of the affected joints (Lautenbach et al., 2013). The RA synovium is enriched with a variety of cell types, including synovial fibroblasts, immune cells (e.g., mast cells, macrophages, neutrophils, dendritic cells, B cells, and T cells; Hitchon and El-Gabalawy, 2011), and newly formed blood vessels from the pre-existing vasculature (angiogenesis; Paleolog, 2002) (Figure 1). Hypoxia is an important micro-environmental characteristic of RA (Lund-Olesen, 1970; Hitchon and El-Gabalawy, 2004; Gaber et al., 2005; Konisti et al., 2012). This is mainly due to hyperplasia of the synovial lining and increased infiltration of immune cells, which together enhance the synovial demand for oxygen (Peters et al., 2004; Lee et al., 2007; Jeon et al., 2008). Induction of angiogenesis is likely a consequence of synovial hypoxia in RA (Paleolog, 2002). However, this increased neovascularization, primarily of dysregulated and immature blood vessels, is unable to provide adequate oxygen perfusion to balance the increased demand (Distler et al., 2004; Taylor and Sivakumar, 2005; Kennedy et al., 2010). Analysis of the oxygen tension (pO_2_) levels in the synovial fluid of knee joints in RA patients have consistently shown significantly lower levels (51.0 ± 16.5 mmHg) in comparison to patients with osteoarthritis (79.2 ± 14.0 mmHg), despite similar synovial thickness scores (Lee et al., 2007). This suggests that synovial proliferation may contribute more to hypoxia in the joint cavities of RA patients.

In response to the alterations in oxygen tension, hypoxia-inducible factors (HIFs) are activated as an adaptive mechanism. HIFs are considered the “master regulators” (Imtiyaz and Simon, 2010), and belong to the family of basic–helix-loop-helix/Per-ARNT-Sim (bHLH/PAS) DNA binding transcription factors (Greer et al., 2012). HIF-1 is a heterodimeric transcription factor, composed of two subunits—HIF-1α and HIF-1β. HIF-1α is regulated by oxygen levels and post-translational modifications that are sensitive to oxygen levels, whereas HIF-1β is expressed constitutively in the nucleus (Konisti et al., 2012). Three HIF-α isoforms have been identified to date (HIF-1α, HIF-2α HIF-3α), with HIF-1α being the most widely investigated (Fan et al., 2014). Activation of the HIF pathway generally allows cells to survive in a hypoxic environment (Semenza, 1998; Harris, 2002), however in some cases these adaptive responses can accelerate disease progression (Ikeda, 2005). Increasing evidence suggest that HIFs play a major role in perpetuating the pathogenesis of RA (Figure 1). In particular, HIF-1α can activate pathways that lead to synovitis, angiogenesis, cartilage degradation, and bone erosion (Konisti et al., 2012; Elshabrawy et al., 2015; Biniecka et al., 2016; Hu et al., 2016). Recent studies have begun to investigate the role of HIF-2α in RA, with initial results demonstrating a potential role as a catabolic factor (Huber et al., 2006; Ryu et al., 2014; Huh et al., 2015). This review will focus on the differential expression of HIF within the synovial joint and its functional behavior in different cell types to regulate disease progression. Potential development of new therapeutic strategies targeting HIF-regulated pathways at sites of disease will also be addressed.

## HIF expression within affected RA joints

HIFs are highly expressed in the hyperplastic RA synovium, which primarily consists of fibroblast-like synoviocytes (FLS) and macrophage-like synoviocytes (MLS; Hitchon et al., 2002). FLS are key effector cells in the pathogenesis of RA, and are crucial in initiating and driving RA together with inflammatory cells (Li et al., 2013a). Studies have reported a patchy and generalized HIF expression throughout the synovial tissue in both the nucleus and cytoplasm (Giatromanolaki et al., 2003; Westra et al., 2010). The degree of expression of the various HIF-α isoforms is only beginning to be elucidated. HIF-1α is reported to be strongly expressed in the intimal layer of the RA synovium, including in resident macrophages (Hollander et al., 2001; Ahn et al., 2008). It has also been detected in the sub-lining layer in lower amounts (Hollander et al., 2001). Conversely, others have found HIF-1α expression to be sparse in the RA synovium and reported HIF-2α as the predominant isoform expressed in human RA joints and in the collagen-induced arthritis (CIA) model (Ryu et al., 2014). In particular, HIF-2α expression was mainly observed in FLS of the RA synovium (Ryu et al., 2014). Furthermore, HIF-1α and HIF-2α have also been identified in resident and infiltrating immune cells (e.g., macrophages, T cell, and mast cells), chondrocytes, and osteoclasts (Westra et al., 2010; Hardy et al., 2014; Ryu et al., 2014). Understanding the expression of HIFs within the RA joint allows us to better understand the degree in which they are activated based on disease severity, as well as how they affect particular cell types to contribute to perpetuating the disease.

## HIF and inflammation

Synovitis is one of the major characteristics of RA. The synovium is a thin and soft tissue lining that consists of the intimal lining (intima) and an underlying loose connective tissue called the sub-lining (sub-intima) layer (Smith, 2011). The architecture of the synovium changes as the disease progresses, resulting in significant tissue edema and hyper-proliferation of the synovial cell lining to 10–15 layers (McInnes and Schett, 2011). The sub-intimal layer becomes heavily infiltrated with immune cells and undergoes neovascularization (McInnes and Schett, 2011). Increasing evidence suggest that HIF-1α acts as a key regulator of inflammation in RA (Eltzschig and Carmeliet, 2011) (Figure 1). For example, PI3 kinase/Akt-mediated HIF-1α expression has been shown to play a critical role in hypoxia-induced epithelial-mesenchymal transition (EMT) phenotype transformation of FLS, synovial hyperplasia, and inflammatory cell infiltration *in vivo* in the CIA model (Li et al., 2013a). Conditional knock-out of HIF-1α in animal models of RA demonstrated clinical and histological improvement of experimental arthritis (Cramer et al., 2003). In particular, there was a significant reduction in synovial inflammation, pannus formation, and cartilage destruction in HIF-1α knock-out mice (Cramer et al., 2003). Inhibition of HIF-1α signaling also attenuated hypoxia-induced invasiveness of activated FLS from the synovium of RA patients (Li et al., 2013b, 2015). Similarly, a recent study reported that HIF-2α itself is capable of producing typical RA characteristics, with HIF-2α deficient mice showing reduced development of experimental arthritis (Ryu et al., 2014).

Inflammatory factors are known to upregulate the expression HIF-1α and HIF-2α in RA. Pro-inflammatory cytokines such as IL-1, TNF-α, and IL-33 have previously been reported to increase the expression of HIF isoforms in synovial fibroblasts (Thornton et al., 2000; Hu et al., 2013; Ryu et al., 2014) and in animal models of RA (Xu et al., 2008; Hu et al., 2013; Ryu et al., 2014). However, limited studies have investigated the direct effect of HIFs in upregulating pro-inflammatory cytokines in RA (Table 1). Recently, Hu et al. reported that HIF-1α was able to directly potentiate the production of inflammatory cytokines (IL-6, IL-8, TNF-α, and IL-1β) and cell-cell contact mediators [IL-15, vascular cell adhesion molecule-1 (VCAM-1), thrombospondin-1 (TSP-1), and stromal cell-derived factor-1 (SDF-1/CXCL12)] in rheumatoid arthritis synovial fibroblasts (RASF; Hu et al., 2016). This was suggested to perpetuate RASF and T-cell/B-cell interactions, leading to RASF-mediated expansion of inflammatory Th1 and Th17 cells, as well as autoantibody production. In particular, knocking down HIF-1α in RASF inhibited IFN-γ, IL-17, and IgG production while enhancing protective natural IgM secretion by T cells and B cells (Hu et al., 2016). Co-expression of SDF-1 with HIF-1α has been identified in both synovial tissue explants and synovial fibroblasts (Hitchon et al., 2002; Hu et al., 2016). SDF-1 is a chemokine that is upregulated in response to hypoxia in RA (Hitchon et al., 2002; Santiago et al., 2011), and is involved in a number of pathogenic events such as increased synovitis, angiogenesis, bone erosion, and cartilage destruction (Villalvilla et al., 2014). Upregulation of HIF-1α has also been shown to significantly enhances the expression of IL-33, which is then able to form a HIF-1α/IL-33 regulatory circuit to further increase HIF-1α expression (Hu et al., 2013). TNF-α converting enzyme, which is involved in regulating TNF-α levels, has also demonstrated to be HIF-1α dependent in RA (Charbonneau et al., 2007). The effect of hypoxia and HIFs in regulating the expression of inflammatory factors is complex, as more than one pathway may be involved in inducing expression. For example, IL-6 expression was significantly increased in FLS under hypoxic conditions, however there are contradictory reports of the influence of HIF-1α on its expression (Ahn et al., 2008; Hu et al., 2016).

**Table 1:** **Direct effects of HIF-α on cells in the RA joint**.

**Cell type**	**Mediator**	**Source(s)**
Synovial fibroblasts	↑ IL-1β	Hu et al., 2016
	↑ IL-6	Hu et al., 2016
	↑ IL-8 (CXCL8)	Ahn et al., 2008; Hu et al., 2016
	↑ IL-15	Hu et al., 2016
	↑ IL-17	Hu et al., 2016
	↑ IL-33	Hu et al., 2013, 2016
	↑ TNF-α	Hu et al., 2016
	↑ TNF-α converting enzyme	Charbonneau et al., 2007
	↑ IFN-γ	Hu et al., 2016
	↑ SDF-1 (CXCL12)	Hu et al., 2016
	↑ MMP-1	Ahn et al., 2008; Lee et al., 2012
	↑ MMP-2	Li et al., 2013c
	↑ MMP-3	Ahn et al., 2008
	↑ MMP-9	Li et al., 2013c
	↓ MMP-13	Lee et al., 2012
	↑ VEGF	Akhavani et al., 2009; Hu et al., 2016
	↑ EMT phenotype transformation	Li et al., 2013a
	↑ VCAM-1	Hu et al., 2016
	↑ TSP-1	Hu et al., 2016
	↑ IgG production	Hu et al., 2016
	↓ Protective natural IgM	Hu et al., 2016
Myeloid cells	↑ TLR-4	Kim et al., 2010
	↑ VEGF	Fava et al., 1994
	↑ CCL20 (MIP-3α)	Bosco et al., 2008
	Regulates glycolysis and energy metabolism	Cramer et al., 2003
	Protects THP-1 cells against TLR-7/8-induced depletion of ATP	Nicholas and Sumbayev, 2009
Chondrocytes	Essential in supporting chondrocyte energy generation	Otero and Goldring, 2007

Toll like receptors (TLR) are pattern recognition receptors that are mainly expressed in immune cells and RASF cells in RA to regulate inflammatory responses (Brentano et al., 2005; Ospelt et al., 2008; Huang and Pope, 2009; Hu et al., 2014a). TLRs can be activated by a number of different factors present within the RA joint (Goh and Midwood, 2012; Xu et al., 2013; Lee et al., 2014). Limited data is available on the regulation of TLRs via hypoxia and HIFs in the pathogenesis of RA (Table 1). Kim et al. showed that hypoxia was able to upregulate the expression of TLR-4 in macrophages via a HIF-1α dependent pathway, leading to the activation of inflammatory mediators in macrophages (e.g., cyclooxygenase-2, IL-6, and RANTES; Kim et al., 2010). Furthermore, HIF-1α may also help in the survival against TLR7/8-induced ATP depletion in THP-1 human myeloid macrophages (Nicholas and Sumbayev, 2009). Overexpression of HIF-1α has been reported to enhance RASF-mediated expansion of inflammatory Th1 and Th17 cells, as well as enhance inflammatory cytokine expression in polyIC-stimulated RASF—thereby inducing a shift toward a pro-inflammatory state in RA (Hu et al., 2014b, 2016). This suggests that hypoxia and HIF-1α may function in conjunction with TLR-stimulated innate immune responses to drive inflammation in RA.

Immune cells also play an important role in synovial inflammation. HIF-1α and HIF-2α are highly expressed in immune cells, in particular macrophages, in the RA synovium (Hollander et al., 2001; Brouwer et al., 2009; Hardy et al., 2014). Resident macrophages (Type A synoviocytes) are primarily located in the intimal lining of the RA synovium, and are also found in the sub-lining layer and at the cartilage pannus junction (Kinne et al., 2000; Szekanecz and Koch, 2007). In response to hypoxia, macrophages upregulate HIFs to activate pathways involved in proliferation, angiogenesis, and glucose metabolism (Hollander et al., 2001; Brouwer et al., 2009; Hardy et al., 2014). Cramer et al. reported a significant reduction in synovitis, pannus formation, and disease progression with HIF-1α knockout myeloid cells in the K/BxN serum transfer arthritis model (Cramer et al., 2003). Deletion of HIF-1α in myeloid cells reduced disease severity in both acute and chronic inflammation. Similarly, macrophages lacking HIF-2α significantly suppressed disease development in the K/BxN serum transfer arthritis model, indicating an important role for HIF-2α in supporting macrophages (Hardy et al., 2014).

## HIF and angiogenesis

Synovial angiogenesis is likely a consequence of synovial hypoxia in RA (Konisti et al., 2012; Azizi et al., 2014). The increased blood supply plays an important role in transporting immune cells to the site of inflammation and supplying nutrients to the pannus (Szekanecz and Koch, 2008). However, this dysregulated vasculature within the RA joint is unable to provide adequate oxygen supply (Taylor and Sivakumar, 2005; Kennedy et al., 2010) and promotes the generation of reactive oxygen species (ROS), which further enhances tissue damage within RA joints (Taylor and Sivakumar, 2005). HIFs have been suggested to regulate the expression of proangiogenic mediators, including vascular endothelial growth factor (VEGF; Fava et al., 1994; Koch et al., 1994), chemokine IL-8 (CXCL8; Ahn et al., 2008), CC-chemokine ligand 20 (CCL20; also known as macrophage inflammatory protein 3α, MIP-3α; Bosco et al., 2008), and SDF-1 (del Rey et al., 2009; Santiago et al., 2011; Table 1). VEGF is a key regulator of angiogenesis that is involved in proliferation, migration, vascular tube formation, and prevention of endothelial cell apoptosis (Elshabrawy et al., 2015). Both HIF-1α and HIF-2α have been shown to increase the expression of VEGF in the inflammatory joint region, as well as in cells derived from RA synovium (Fava et al., 1994; Koch et al., 1994; Konisti et al., 2012). It should be noted that other pathways dependent or independent of HIF may also be involved in regulating the expression of proangiogenic factors. For example, the Notch signaling pathway has been shown to mediate hypoxia-induced angiogenesis in inflammatory arthritis via the interaction of Notch-1/HIF-1 through VEGF/Angiopoietin-2 (Gao et al., 2012) In addition, pro-inflammatory mediators are also able to promote RA angiogenesis, including cytokines [e.g., IL-17, IL-18, and macrophage migration inhibitory factor (MIF)], chemokines (CXCL12), growth factors (e.g., Ang1 and Ang2), proteases (MMPs), and adhesion molecules (e.g., ICAM-1 and VCAM-1; Elshabrawy et al., 2015).

## HIF and cartilage destruction

The articular cartilage is an avascular, aneural, and alymphatic tissue, which is composed primarily of chondrocytes. The role of HIFs in cartilage destruction in RA is not yet fully characterized, with the majority of the work conducted in healthy articular cartilage. Cells in the articular cartilage normally reside in a hypoxic environment, with oxygen tension varying from 6–10% at the joint surface to 1% in the deeper layers (Gibson et al., 2008). HIF-1α acts as a survival factor in healthy cartilage (Schipani et al., 2001; Pfander et al., 2003; Mariani et al., 2014), and is essential in supporting chondrocyte energy generation (Pfander et al., 2003, 2005; Mobasheri et al., 2005; Mariani et al., 2014) and synthesis of the cartilage matrix (Pfander et al., 2005; Mariani et al., 2014). Similarly, HIF-2α has been shown to have a major role in anabolic responses in healthy human chondrocytes (Lafont et al., 2007, 2008; Thoms et al., 2013; Zhang et al., 2015). Initial results suggest that HIF may be involved in perpetuating cartilage destruction in RA (Figure 1). As chronic inflammation advances in RA, the hyperplastic pannus aggressively invades and destroys the articular cartilage, particularly in the regions that are contiguous with the proliferating synovial pannus (Williams et al., 2000; Goldring, 2003; Otero and Goldring, 2007). The synovial pannus contains invasive RASF cells which are the main cells responsible for marginal cartilage destruction, via the production of cartilage degrading enzymes called matrix metalloproteinases (MMPs) (Abeles and Pillinger, 2006). Recent studies have shown that membrane-type MMPs (MT1-MMP and MT3-MMP) (Pap et al., 2000a,b) and cathepsin group of proteases (cathepsin B, cathepsin L, capthesin D and capthesin K) also contribute to cartilage destruction in RA (Pap et al., 2000b).

Hypoxia has been demonstrated to upregulate the levels of MMPs in cells derived from RA synovium, although additional pathways independent of HIF may also be involved (Canning et al., 2001; Ben-Yosef et al., 2002; Cha et al., 2003; Akhavani et al., 2009; Table 1). Lee et al. demonstrated an increase in MMP-1 protein expression and a corresponding decrease in MMP-13 protein expression in IL-1β-stimulated FLS derived from RA synovium under hypoxic conditions (Lee et al., 2012). This differential effect of hypoxia on MMP production was shown to be HIF-1α-dependent. MMP-3 has also been shown to be directly controlled by the activation of HIF-1α in FLS (Ahn et al., 2008). Interestingly, hypoxia-induced MMP-1 expression was not significantly attenuated by knock-down of HIF-1α, which suggests other pathways are also involved in the expression of MMPs (Ahn et al., 2008). Similarly, IL-17A was shown to induce MMP-2 and MMP-9 expression, via a HIF-1α/NF-kB pathway (Li et al., 2013c). Although the majority of the initial studies have been conducted *in vitro* on cells derived from RA synovium, further *ex vivo* and *in vivo* studies are warranted to assist in strengthening our understanding of the complex role of HIF in cartilage degradation in RA.

## Targeting HIF-regulated pathways in RA

Studies to date suggest that HIFs are promising targets for novel RA treatments. Approaches that may be considered for targeting hypoxia in RA cells include the use of hypoxia-activated prodrugs, specific HIF inhibitors, gene therapy, or targeting indirect pathways important in hypoxic cells. The majority of these targeted therapies have come from research on the effects of hypoxia on the growth of tumors (Phillips, 2016; Wigerup et al., 2016). Hypoxic prodrugs were designed to be selectively activated in hypoxic tissue, via reduction of the prodrug by cellular reductases, thereby delivering the active agent to hypoxic cells (Phillips, 2016). These therapeutic agents appear to use hypoxia as a targeting mechanism to deliver therapeutic agents to specific disease sites. The main issue is the risk of off-target effects due to the fact that hypoxia is a dynamic process that occurs physiologically as well as in a multitude of diseases.

A number of HIF inhibitors have been developed that possess inhibitory activity against cancer and HIF-related diseases (Ban et al., 2016). There are considerably fewer selective and specific HIF inhibitors compared to non-selective HIF inhibitors that can target multiple pathways. Initial clinical trials of these agents in various forms of cancer and other hypoxia-related diseases have shown encouraging results (Konisti et al., 2012; Wigerup et al., 2016). The majority of these compounds are still in the early stages of development and have yet to be assessed in clinical trials for RA (Manabe et al., 2008; del Rey et al., 2009; Shankar et al., 2009; Wigerup et al., 2016). Reasons for this include the complexity of the HIF pathway in RA and their different mechanisms of action for HIF inhibition, as well as pharmacokinetic and stability issues that prevent these therapeutic agents from reaching the target cells following administration (Ban et al., 2016; Wigerup et al., 2016). Local administration of these compounds (e.g., intra-articular injection) was proposed as an option to avoid any pharmacokinetic issues or premature systemic degradation. However, this route of administration is not always preferable in conditions involving multiple sites and/or difficult to reach sites.

The use of delivery carriers may improve the efficacy of HIF-related therapeutic agents following systemic administration, by overcoming the pharmacokinetic and stability issues. The best example of this is in the delivery of gene therapy targeting HIFs, which have previously been shown to modulate cellular responses in hypoxia-related diseases (Post et al., 2007; Tal et al., 2008; Wang et al., 2008; del Rey et al., 2009; Chen et al., 2016). The delivery of small interfering RNA (siRNA) remains a challenge because of the lack of suitable vectors. Transfection of fibroblasts with lentiviruses expressing HIF-1α siRNA demonstrated significant reduction in HIF-1α accumulation and VEGF mRNA levels in an *in vivo* model of RA (del Rey et al., 2009). Although viral vectors have shown to be effective, the concerns surrounding their safety have limited their application. Therefore, non-viral vectors have been investigated as an alternative delivery platform for HIF gene delivery—including liposomes (Wang et al., 2008; Chen et al., 2016) and polymer-based nanoparticles (Liu et al., 2009). For example, Wang et al. demonstrated that PEGylated liposomes loaded with doxorubicin and antisense oligonucleotides targeted to HIF-1α mRNA was able to effectively deliver the active agents into tumor cells on nude mice bearing xenografts of multidrug-resistant human ovarian carcinoma (Wang et al., 2008). The proposed combination therapy increased the therapeutic efficacy of the chemotherapy to an extent that was not able to be achieved by administration of the individual components separately (Wang et al., 2008). These results demonstrate that packaging active agents targeting HIF-regulated pathways into delivery vehicles, which are designed to accumulate specifically at sites of RA disease, may improve therapeutic efficacy and reduce off-target effects (Sercombe et al., 2015).

Indirect strategies to target downstream HIF signaling pathways have also been investigated in RA. For example, many therapeutic approaches have successfully resolved angiogenesis in preclinical animal models of RA by administering antibodies targeting VEGF or small molecule inhibitors targeting the VEGF receptor (Maruotti et al., 2014). De Bandt et al. demonstrated that systemic administration of an anti-VEGF-RI monoclonal antibody (mAb) in the K/BxN mouse model of RA was able to delay the onset of arthritis, attenuate its intensity, and prevent joint destruction (De Bandt et al., 2003). Interestingly, Lu et al. reported that administration of anti-VEGF antibody before the onset of experimental arthritis was able to delay the onset and reduce the severity of disease in the CIA model, whereas administration post-onset of disease had no effect on the progression or severity of the arthritis (Lu et al., 2000). The study suggested that VEGF may have a crucial role predominantly in the early stage of arthritis development. Unfortunately, a number of these promising therapies have not yet been tested in clinical trials for RA. Those which have been tested in clinical trials were unable to translate the promising results in preclinical studies into successful treatment strategies for RA patients. This includes the discontinuation of phase II clinical trials studying MMP inhibitors in RA (Thabet and Huizinga, 2006; Dorman et al., 2010), as well as reduced efficacy of repeated administration of anti-ICAM-1 mAb in RA patients (Kavanaugh et al., 1996, 1997). This suggest that human RA is highly complex and heterogeneous, which involves multiple pathways occurring simultaneously to perpetuate disease (Elshabrawy et al., 2015).

## Conclusion

Accumulating evidence supports hypoxia and HIFs in regulating a number of important pathophysiological characteristics of RA, including synovial inflammation, angiogenesis, and cartilage destruction. Therefore, HIF inhibitors are likely to target multiple important RA processes. Experimental and clinical data have confirmed the upregulation of both HIF-1α and HIF-2α in RA. At this time, the relative importance of each isoform in RA pathology and disease severity is still unclear. These two isoforms show different sensitivity to oxygen tension and display distinct, and sometimes opposing, cellular activities. In addition, further studies are required to clarify the interrelationship between HIFs and other simultaneous pathways in perpetuating RA disease. This will allow us to determine whether specific HIF-1α or HIF-2α inhibition is likely to be required for successful clinical outcomes in RA. To optimize their effects, HIF inhibitors may require encapsulation within delivery vehicles directed toward affected RA tissue to improve therapeutic accumulation and stability following systemic administration, as well as reduce off-target effects.

## Author contributions

SH was responsible for assembling, drafting and revising the manuscript, and preparing the associated figure and table. TD contributed to the drafting of this manuscript.

### Conflict of interest statement

The authors declare that the research was conducted in the absence of any commercial or financial relationships that could be construed as a potential conflict of interest.
